# Hydrocele in a case of atypical Kawasaki disease: case report and review of diagnostic criteria

**DOI:** 10.1186/s12887-021-02758-1

**Published:** 2021-06-15

**Authors:** Y. R. L. Tan, C.-T. C. Chow, I. Ganesan, H. M. E. Leow

**Affiliations:** Department of Paediatrics, KK Women’s and Children’s Hospital, 100 Bukit Timah Road, Singapore, 229899 Singapore

**Keywords:** Case report, Kawasaki disease, Atypical, Diagnostic criteria, Hydrocele

## Abstract

**Background:**

Kawasaki Disease (KD) is a self-limiting vasculitis of unknown etiology. Although there are well-recognized clinical features associated with classic KD, there have been increasing numbers of atypical clinical presentations with increased dependence on the American Heart Association diagnostic algorithm for incomplete KD.

**Case presentation:**

We report on a child who was initially treated for *Escherichia coli* left pyelonephritis and Influenza A and Rhinovirus / Enterovirus upper respiratory tract infection. The child developed an acute hydrocele and a maculopapular rash during the illness course, which prompted further evaluation for concomitant atypical KD, although there were no other physical signs suggestive of classic KD at the time. Subsequent diagnosis of atypical KD was made with confirmation on echocardiography, with timely administration of intravenous immunoglobulin.

**Conclusions:**

Although there are well recognized clinical features associated with classic Kawasaki Disease, there have been increasing numbers of atypical clinical presentations with increased dependence on the American Heart Association diagnostic algorithm for incomplete Kawasaki Disease. This case report highlights the importance of considering a diagnosis of KD in a child with prolonged fever and unexplainable symptoms suggestive of inflammation, in this case, the rare presentation of an acute hydrocele. We recommend that for any child with prolonged unexplained fever, Kawasaki Disease should be considered.

**Trial registration:**

Not applicable.

## Background

Kawasaki disease is a self-limiting vasculitic syndrome that predominantly affects medium and small-sized arteries and can be complicated by the development of coronary artery aneurysms (CAA) in 25% of untreated patients, which fall to 4% with treatment [1]. It is the leading cause of acquired paediatric heart disease worldwide, and can lead to myocardial infarction and late coronary artery stenosis [1]. It tends to affect previously healthy infants and children. Accurate diagnosis of incomplete and atypical Kawasaki Disease is important, because these patients are at risk of coronary artery lesions seen in typical Kawasaki Disease [2].

Classic Kawasaki Disease as defined by the American Heart Association (AHA) is associated with fever of at least 4-day duration and clinical features such as conjunctivitis, rash, adenopathy, mucositis and oedema of extremities [3], but there have also been increasing numbers of case reports of unusual clinical presentations of Kawasaki Disease, such as gallbladder hydrops and acute cholestatic hepatitis [4].

In this case report, we describe a patient with acute testicular hydrocele who was subsequently diagnosed to have incomplete Kawasaki Disease. We wish to highlight the importance of considering a diagnosis of Kawasaki Disease in a child with prolonged fever and unexplainable symptoms suggestive of inflammation, especially unexpected presentations such as a testicular hydrocele, as timely diagnosis is essential to reduce treatment delay.

### Visit summaries

The child was admitted to the Paediatric Medicine department in Singapore’s KK Women’s and Children’s Hospital (KKH) from January to February 2020 for 16 days. He was subsequently followed up in the nephrology outpatient clinics for four further times – 1, 3, 6 and 10 months post discharge. He also followed up in the cardiology outpatient clinic – 3 and 6 months after discharge.

### Case presentation

An 18-month-old boy of Chinese ethnicity, with a previous urinary tract infection at 4 months of age and complex febrile seizure at 9 months of age, was admitted with a 4-day duration of fever, vomiting and cough, with daily temperatures of ≥38 °C. Other than nasal congestion, his physical examination on presentation was unremarkable. Vital signs recorded were within normal limits for his age. On admission, C-reactive protein (CRP) was significantly elevated at 294.4 mg/L and total white blood cell count (TW) was slightly elevated at 14.92 ×  10^9^/L. Urinalysis done showed pyuria and he was started on intravenous ceftriaxone at the dose of 50 mg/kg/day. Urine cultures grew pan-sensitive *Escherichia coli*, and aerobic blood culture returned sterile. Despite appropriate antibiotic coverage, he continued to have persistent fever, with the daily temperature reaching up to the highest of 41.3 °C on day 5 of fever. The dose of ceftriaxone was optimized to 100 mg/kg/day on day 7 of fever, and a referral to the Paediatric Nephrologist was made. Ultrasound of the kidney, bladder and ureters (US KUB) performed on day 6 of fever showed left focal pyelonephritis, with subsequent repeat US KUB on day 11 showing hypoechoic foci within the lateral aspect of his left kidney indicative of small abscesses (largest measuring 0.8 cm). There was no hydronephrosis or hydroureter noted. Subsequent repeat urine cultures performed on day 9 and 16 of fever returned sterile. Nasopharyngeal aspirate performed on day 7 of fever returned positive for Influenza A and Rhinovirus / Enterovirus infection, for which he was treated symptomatically.

**Fig. 1:**
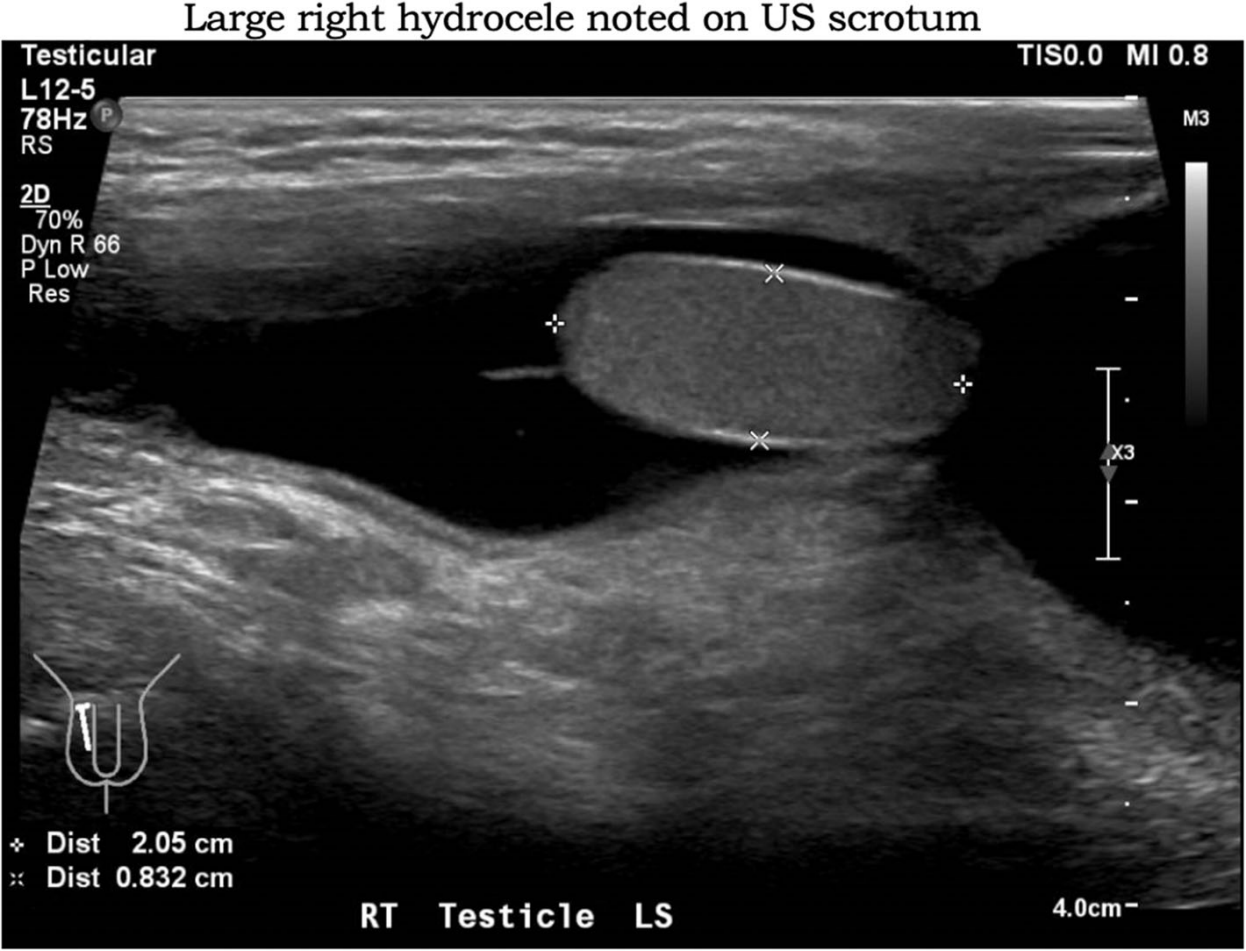
Large right hydrocele notes on US scrotum

He developed a sudden onset of right scrotal swelling on day 8 of fever. Ultrasound scrotum performed showed a large right hydrocele (Fig. 1) and a small right testis appendix, with no focal abnormalities noted in left testis or the epididymis. The right hydrocele extended into the inguinal region, in-keeping with a patent processus vaginalis. No whirlpool sign was seen bilaterally. The Paediatric Surgeon opted to manage the child conservatively, as there was no clinical or sonographic evidence of epididymal torsion or infection.

He also developed a generalized maculopapular rash over the trunk and bilateral lower limbs on day 8 of fever. He did not have other new physical examination findings. In view of prolonged fever despite appropriate antibiotics, new onset of rashes and testicular hydrocele, laboratory studies were sent off on day 9 of fever, with Tables 1 and 2 showing the timeline of blood investigations and microbiological investigations performed.

**Table 1:** Timeline of Blood Investigations Done

	Day of Illness				
	Day 4	Day 6	Day 9	Day 11	Day 16
Hemoglobin (g/dL) (12.0–15.0)	10.8	10.6	10.5		9.8
White blood cell count (× 10^9^/L) (6.00–17.50)	14.92	7.50	8.67		9.22
Platelet count (× 10^9^/L) (140–440)	210	179	298		620
Erythrocyte sedimentation rate (mm/h) (1–10.0)			91		
C-reactive protein (mg/L) (0–5.0)	294.4	156.2	90.2	37.8	5.8
Albumin (g/L) (35–45.0)			26		
Alanine transaminase (U/L) (9–25.0)			19		
Aspartate transaminase (U/L) (21–44.0)			28		
Alkaline phosphatase (U/L) (166–393.0)			133		
Gamma-glutamyl transferase (U/L) (6–15.0)			28		
Sodium (mmol/L) (138–145.0)	130		134

**Table 2:** Timeline of Microbiological Investigations Done

	Day of Illness			
	Day 4	Day 7	Day 9	Day 16
Nasopharyngeal aspirate immunofluorescence	Negative			
Nasopharyngeal aspirate multiplex		Rhinovirus / Enterovirus and Influenza A positive		
Blood culture (aerobic)	Negative			
Urinalysis	RBC 108/uL, WBC 572/uL, Nitrite NEG, Leu 3+, Protein 2+ (catheterized urine sample)		RBC 2/uL, WBC 37/uL, Nitrite NEG, Leu NEG, Protein 1+ (clean catch urine sample)	RBC 0/uL, WBC 3/uL, Nitrite NEG, Leu NEG, Protein 1+ (clean catch urine sample)
Urine culture	*E. coli* (Pan sensitive), viable count > 100,000 colony-forming units (CFU) (catheterized urine sample)		Negative (clean catch urine sample)	Negative (clean catch urine sample)

Based on the American Heart Association (AHA) 2017 scientific statement of the diagnosis and management of KD [3], he had the following laboratory readings supportive of Kawasaki Disease – normocytic normochromic anemia, hypoalbuminemia, raised CRP and Erythrocyte Sedimentation Rate (ESR). However, he only demonstrated two clinical features of classic KD: prolonged fever and rash.

The child was treated as for atypical Kawasaki Disease, and one dose of intravenous immunoglobulin (IVIg) 2 g/kg was given on day 9 of fever, and oral aspirin 5 mg/kg/day was started on the same day. The fever resolved within 36 h of completion of the IVIg infusion.

Diagnosis of KD was subsequently confirmed by echocardiography performed on day 14 of illness, with the following results: top normal left coronary artery; mildly dilated left anterior descending artery (LAD); normal right coronary artery (RCA) proximally with no dilatation or aneurysm visualized; left main coronary artery (2.63/2.70/2.76 mm, 2.70 mm (mean), Z-score: + 2.3 (Kobayashi), + 4/5 (Tan-KKH)); LAD (2.36/3.28/2.41 mm, 2.68 mm (mean), Z-score: + 2.9 (Kobayashi)); RCA (2.34/2.12/2.25 mm, 2.24 mm (mean), Z-score: + 1/9 (Kobayashi), + 3/7 (Tan-KKH)) [5, 6].

At 6 weeks post-discharge, US KUB showed resolution of suppurative changes after a total of 6 weeks of antibiotics, and repeat US scrotum showed complete resolution of right hydrocele with normal sized testes bilaterally. Repeat 2DE in 3 months outpatient showed resolution of dilatation of left anterior descending artery noted previously. Aspirin was stopped in outpatient cardiology clinic on review of 2DE in view of good improvement. Micturating cystourethrography done in 4 months post discharge showed mild dilatation of ureter and pelvicalyceal system, with mild blunting of the fornices, indicative of grade III left vesico-ureteric reflux. Dimercaptosuccinic acid (DMSA) scan done in 7 months post discharge showed focal photopenia in upper pole of left kidney suggestive of cortical scarring, with differential renal function of 38.53% on left and 61.47% on the right. The child is continued on cefalexin uroprophylaxis dose of 17 mg/kg once nightly.

### Diagnostics

#### Patient perspective

This patient’s parents were concerned and worried about the cause of his unremitting fever. They were also distressed by their child’s persistent irritability and poor feeding during his prolonged hospitalisation. After extensive discussion about the possibility of Kawasaki Disease, they were agreeable for treatment with intravenous immunoglobulin. When 2DE results were consistent with Kawasaki Disease after timely administration of intravenous immunoglobulin, his parents were thankful to the clinicians who considered this differential which led to prompt treatment of the disease.

## Discussion and conclusions

The current understanding of Kawasaki Disease pathophysiology is limited due to several factors – low availability of human tissues of disease, failure to identify specific etiological triggers, and incomplete understanding of underlying molecular and cellular mechanisms [7]. In a post-mortem study of patients with Kawasaki Disease, 73% of patients were identified to have renal artery involvement and acute kidney injury involving glomerulonephritis with intracapillary changes and deposition of immune complex composed of IgA and complement component 3 [8]. These findings are comparable to findings of other inflammatory human diseases like IgA vasculitis and IgA nephropathy [8]. This suggests that the disease is largely associated with inflammation.

Because of the wide range of clinical presentations, epidemiological surveys were used to develop clinical diagnostic criteria for the disease. Epidemiological surveys from Japan were used to produce a set of diagnostic criteria [1, 9], and this is also reflected in the latest AHA 2017 scientific statement by McCrindle et al. [3]:

(a) Fever of at least 5 days duration, with at least 4 out of the 5 criteria listed below, in the absence of another known disease process to explain the illness

(i) Erythema and cracking of lips, strawberry tongue, and/or erythema of oral and pharyngeal mucosa
(ii) Bilateral bulbar conjunctival injection without exudate
(iii) Rash: maculopapular, diffuse erythroderma, or erythema multiforme-like
(iv) Erythema and oedema of the hands and feet in acute phase and/or periungal desquamation in subacute phase
(v) Cervical lymphadenopathy (≥1.5 cm diameter), usually unilateral

Patients with fewer than 4-out-of-5 of the principal symptoms can be diagnosed with Kawasaki Disease when coronary aneurysm or dilatation is diagnosed by echocardiography or coronary angiography [3]. However, diagnosis remains a challenge, as the defined criterion for Kawasaki Disease does not identify all children with the illness [3]. Many children with Kawasaki Disease exhibit other reported signs not part of the clinical diagnostic criteria, including irritability and inflammation at the site of a previous BCG immunization [10]. Some other less common but associated clinical findings include arthritis, aseptic meningitis, pneumonitis, uveitis, gastroenteritis, meatitis, dysuria and otitis [11]. Rarer abnormalities include gallbladder hydrops [4], gastrointestinal ischemia, jaundice, petechial rash, febrile convulsions, encephalopathy, ataxia, syndrome of inappropriate antidiuretic hormone secretion and macrophage activation syndrome [11, 12]. Due to its wide range of clinical presentations, Kawasaki Disease may manifest as a diagnostic dilemma [13]. It was especially difficult in our case, as the raised inflammatory markers (CRP, ESR) may be attributed to the culture proven urinary tract infection. Additionally, prolonged fever may occur with micro-abscesses in the kidney, and rash is a common finding in viral infections. However, the development of an acute hydrocele, along with clinical evidence of systemic inflammation, prompted the evaluation of atypical Kawasaki Disease, of which the AHA criteria was subsequently fulfilled. This clearly highlights that there is still merit in consideration of Kawasaki Disease, despite having already identified underlying infections that have been adequately and appropriately treated. Therefore, we recommend that for any child with prolonged unexplained fever, especially with other clinical signs suggestive of inflammation, Kawasaki Disease should be considered, even if an infective process or cause of the fever has already been identified, so as to avoid significant delays in diagnosis and delayed intravenous immunoglobulin administration.

The diagnosis of atypical Kawasaki Disease in our case is confirmed by 2DE. The Japanese Ministry of Health (JMH) criterion for Kawasaki Disease coronary involvement is defined on the basis of absolute dimension of internal diameter of coronary artery [14]. The American Heart Association (AHA) 2017 guidelines is adapted from McCrindle et al. [3] and Manlhiot et al’s [15] proposition of a classification scheme based on z score for severity of coronary artery abnormalities. JMH defines aneurysm in children less than 5 years old to have internal diameters (ID) of > 3 mm, with updated JMH guidelines in 2008 defining small aneurysms as ID < 4 mm, medium aneurysms as ID 4-8 mm, and large aneurysms as ID > 8 mm [16]. AHA 2017 revised criteria lists “no involvement of coronary artery” as Z score < 2, “dilation only” as Z score 2 to < 2.5, “small aneurysm” as Z score > =2.5 to < 5, “medium aneurysm” as Z score > =5 to < 10, and “large or giant aneurysm” as Z score > =10 [16]. Other 2DE findings of KD include coronary artery ectasia, dilatation and aneurysm, lack of tapering of coronary arteries, myocardial dysfunction, pericardial effusion, aortic root dilatation and valvular regurgitation [16]. In our case, although the 2DE shows ID ranging from mean of 2.24 to 2.70 mm which does not fulfil JMH criteria, the Z-score of + 2.9 in the LAD is in keeping with AHA 2017 definition of small aneurysm.

There have been previous reports on hydroceles with cases of patients with Kawasaki Disease [17–19]. Kabani et al. described three patients with atypical Kawasaki Disease and communicating hydrocele [18], and Sacco et al. described an additional case of hydrocele that developed at onset of typical Kawasaki Disease [19]. A hydrocele can be produced by excessive fluid production within the scrotal sac, defective absorption of fluid, interference with lymphatic drainage of the testicle and scrotum, or by a direct connection with the peritoneal cavity. The majority of hydroceles present at birth, and are due to the presence of a patent processus vaginalis, which usually close spontaneously by 12 months [20]. The mechanism of sudden processus vaginalis opening in acute Kawasaki Disease remains unknown [17], although it is postulated that the inflammatory process of Kawasaki Disease can contribute to this process. This is supported by case reports of hydroceles developing in testicular infections [21], with Chesney et al. describing a small hydrocele secondary to *Haemophilus influenza* type B [22]. Jibiki et al. have nicely summarized case reports into a table, demonstrating the association of Kawasaki Disease with acute scrotum [17]. This suggests that although the incidence of acute scrotum in patients with Kawasaki Disease is unknown, careful observation may identify additional patients with Kawasaki Disease [17].

In a 2014 study by Xu et al. [23] that studied the trend of C-reactive protein (CRP) in paediatric patients with urinary tract infections pre- and post- antibiotic treatment after 24 h, the pre- and post- treatment CRP levels were 68.17 +/− 39.42 and 26.13 +/− 14.15 mg/L respectively. The significant difference in CRP before and after treatment in the study (*P* < 0.05) [23] shows that CRP can be used for observing pathogenesis and curative effect. In our patient, CRP downtrended at a slower rate from 294.4 mg/L on day 4 to 90.2 mg/L on day 9, suggesting the possibility of a separate ongoing inflammatory process.

The child in our case only had 1 additional compatible clinical criterion (rash) in addition to fever of more than 5 days duration. However, in view of the presentation of an acute hydrocele, further investigations were conducted to evaluate for Kawasaki Disease. This eventually led to the diagnosis of incomplete Kawasaki Disease, hence facilitating the timely administration of intravenous immunoglobulin prior to day 10 of fever.

Although there are well recognized clinical features associated with classic Kawasaki Disease, there have been increasing numbers of atypical clinical presentations with increased dependence on the American Heart Association diagnostic algorithm for incomplete Kawasaki Disease. Timely diagnosis of Kawasaki Disease can reduce cardiovascular morbidity, thus it is important to consider this diagnosis in a child with prolonged fever and unexplainable inflammatory symptoms. We recommend that for any child with prolonged unexplained fever, Kawasaki Disease should be considered, even if an infective process or cause of the fever has already been identified. Acute hydrocele can also be a clinical feature of Kawasaki Disease, and is possibly related to the underlying systemic inflammatory processes, thus clinicians dealing with diagnostic dilemmas involving inflammatory processes should always consider Kawasaki Disease as a potential differential diagnosis.

## Data Availability

All data generated or analysed during this study are included in this published article. Please refer to reference list for citations.

## Abbreviations

AHA: American Heart Association
ALT: Alanine transaminase
CAA: Coronary artery aneurysm
CRP: C-reactive protein
ESR: Erythrocyte sedimentation rate
FBC: Full blood count
hpf: High powered field
LAD: Left anterior descending artery
RBC: Red blood cell
RCA: Right coronary artery
KD: Kawasaki Disease
TW: Total white cell count
US: Ultrasonography
US KUB: Ultrasound of Kidneys, Ureters and Bladder
WBC: White blood cell
2DE: Chocardiography
